# A fuzzy based chicken swarm optimization algorithm for efficient fault node detection in Wireless Sensor Networks

**DOI:** 10.1038/s41598-024-78646-2

**Published:** 2024-11-11

**Authors:** B Nagarajan, Santhosh Kumar SVN, M Selvi, K Thangaramya

**Affiliations:** 1 School of computer Science Engineering and Information Systems, Vellore Institute of Technology, Vellore, India; 2 School of Computer Science and Engineering, Vellore Institute of Technology, Vellore, India

**Keywords:** Node fault, Poisson Hidden Markov model, Optimization, Fault detection, Sensor node, Energy science and technology, Engineering, Mathematics and computing

## Abstract

Wireless Sensor Networks (WSN) are built with miniature sensor nodes (SN), which are deployed into the geographical location being sensed to monitor environmental conditions, which transfer the sensed physical information to the base station for further processing. The sensor nodes frequently experience node failure as a result of their hostile deployment and resource limitations. In WSN, node failure can cause a number of issues, namely Wireless Sensor Networks topology changes, broken communications links, disconnected portions of the network, and data transmission errors. An important concern of WSN is the detecting, diagnosing and recovering of sensor node failures. In the course of this effort, an effective strategy for sensor node failure detection algorithm using the Poisson Hidden Markov Model (PHMM) and the Fuzzy-based Chicken Swarm Optimization (F-CSO) is proposed for efficient detection of sensor node faults in the WSN. The proposed work offers optimal false alarm, false positive, energy consumption, detection accuracy, network lifetime, and least delay rates. Moreover, the F-CSO provides improved localization to locate the defective sensor nodes that are present in the WSN. The proposed work is implemented in the NS2 simulator with realistic simulation parameters, and the simulation results demonstrate that the proposed work is more effective in terms of 89.5% fault detection accuracy, 19.53% throughput, 8.43% energy consumption with minimum delay and less false positive rate when it is compared with other existing state-of-art systems.

## Introduction

WSN is made up of a decentralized collection of numerous, sparsely spaced-out, small-sized sensors with limited computing facilities, low battery power and limited memory for data storage and processing^1^. WSN has been widely employed in many sectors, including the military, healthcare, agriculture, and environmental monitoring^2^. Wireless sensor networks, however, are the most difficult technologies to use since they employ a large number of small sensor devices that are susceptible to failures because of inadequate battery backup, placement of sensor nodes in unattended areas and other undesirable environmental conditions^3^. The sensor devices are impacted by hardware and software problems as a result of environmental threats such as severe downpours, floods, strong winds, and forest fires in WSN^4^. The sensor nodes of WSNs perform the essential tasks such as senses and monitors the environmental conditions, broadcasts those information to the base station or others nodes in the network and relaying. To extend the battery life of sensor nodes is through routing techniques^5–7^.

The probability of node failure is directly proportional to the scalability of the WSN. The SNs are vulnerable to faults because of energy exhaustion, physical damage, communication link failures, and software or hardware failures. The faulty SN is impacted, unable to send data to the base station or to the receiver^8,9^. Therefore, it’s critical to accurately identify the faults in sensor nodes to make SNs perform unlimited services. The failure of SNs in the network results in poor network performance, partitioning of the sensor network, and failure to transmit accurate information to the base station. Moreover, most of the existing fault detection systems suffer from computational overhead on sensor nodes, which makes them vulnerable to early sensor node failure. The existing fault detection techniques, however, have a high false alarm rate and poor fault detection accuracy^10^. Additionally, they use a tremendous quantity of power to identify faulty sensor nodes, which causes the earlier death of SNs^11,12^. Hence, an efficient fault detection approach for WSNs is needed to optimize and have better fault detection accuracy.

Failures of a hardware component, physical harm, low battery, or adverse environmental factors are a few causes of sensor node failure. As a result, the failure can be managed by creating distinct fault detection for various sensor fault types. The sensor node faults are mainly categorized into hard sensor faults and soft sensor faults^13,14^. In hardware-based concerns, faulty sensor nodes are detached from the network and unable to connect and communicate with some other network nodes. In hardware sensor faults will occur when hardware module failures, such as the battery depletion, sensor unit, localization unit, processing unit, microcontroller unit or transceiver unit, cause a hard fault. Software-based sensor failures take place when there is a fault in nodes software, such as malfunction, transmit inaccurate data, or interact incorrectly with other nodes. While hard faults are permanent errors that need replacement of the sensor node, whereas software-based faults can be fixed by changing the current sensor algorithms when they are running in the sensor nodes.

According to fault severity, sensor node defects are further divided into permanent fault, transient fault, and intermittent fault^15^. A sensor module with a permanent defect remains inactive for the duration of its life. Since the problem is ongoing, the component needs to be changed. The sensor generates fault behaviour over a longer span of time, despite the fact that the intermittent fault is not continuous. It is treatable and, after some time, may resume its flawed behaviour. The transient fault is a short-period malfunction that can be automatically corrected. It is exceedingly challenging to diagnose and control transitory errors because they can arise as a result of brief environmental changes^16^. Depending on the network components, WSN failures are even further classified into three distinct categories: network, node, and base station faults. Hardware or software malfunctions led to node defects. A failure node may deviate from initial values and provide incorrect information due to energy depletion and a drop in energy level below the threshold value. Failure of a communication route or link will result in a network fault. These faults lead to issues including poor communication, data packet loss and network failure as a whole. The entire sensor network fails when a sink or base station goes down.

Based on the fault detection and diagnosis strategy, there are three fault management approaches, namely, distributed, centralized and hybrid^17^. Every node in the network shares the process of monitoring and analysis when utilizing distributed approach. In contrast, in a centralized approach, a node with huge battery power and memory is responsible for fault detection and classification. However, the centralized approach relies on a BS or a central node to oversee and assess the health status of other nodes. Hybrid approaches create a multi-tiered WSN architecture that integrates the elements of both distributed and centralized approaches.

The residual nodes of WSN can shorten the lifespan of the network. In order to investigate the environment and analyze the gathered data, resource-constrained SNs have been integrated into specific networks, utilizing one or more gateways. The network gateways could be implemented in a systematic fashion to facilitate communication between WSN sensors to transmit and process data^18,19^. Proper deployment of sinks and SNs is crucial for energy-saving and enhanced network performance. In order to detect and diagnose the faulty nodes in the network, the localization of faulty nodes is another essential operation to provide fault-free communication between SNs and maintain an efficient routing process. Table 1 provides the abbreviations and short forms that are used in the proposed work.

**Table 1:** Abbreviations used in this proposed work.

Abbreviations	Acronyms
Poisson Hidden Markov Model	PHMM
Fuzzy based Chicken Swarm Optimization	F-CSO
Wireless Sensor Networks	WSN
Adaptive Neuro Fuzzy Inference System	ANFIS
Sensor Node	SN
Multi-Fault Detector	MFD
Cellular Learning Automata Faulty Node Detection & Management	CLAFNDM
Faulty Node Classification & Management	FNCM
Fault Management Framework for Markov Chain	FMMC
Optimal Emperor Penguin Optimization	OPEO
Trajectory Pattern Extraction	TPE
reactive Distributed Fault Detection	rDFD
Harmony Search Algorithm	HAS
Medium Access Control	MAC
Received Signal Strength	RSS
Acknowledgement	ACK
Gravitational Search algorithm & Particle Swarm Optimization	GSAPSO
Fuzzy one Class Support Vector Machine based Fault Detection	FCS-MBF
Quality of Service	QoS
Fault Detection Scheme	FDS
Neural Networks	NN
Least-square Support Vector Machine	LSVM
Fuzzy Density-based Spatial-clustering Application with Noise	FDSCAN
Artificial Intelligence	AI
Deep-reinforcement Learning	DRL
Back-Propagation Neural Network	BPNN

Motivating from all these observations in this work, an efficient fault detection scheme is proposed that efficiently detects the sensor node’s failure. The overall contributions of this work are.

- This work presented a fault detection mechanism for both the soft and hard sensor fault detection, which considerably improving the accuracy rate of fault detection while minimizing the false alarms.
- This proposed work employs an efficient fault detection approach, namely the Poisson Hidden Markov Model, which is a probability-based mechanism for efficient fault detection sensor nodes.
- An optimization algorithm, namely Fuzzy-based Chicken Optimization localization algorithm, is proposed for providing improved energy efficiency, fault detection rate, network lifespan, and minimizing false positive and false alarm rates.

The remaining sections of this work are divided into the following sections: Sect. 2 provides insight studies into the types of sensor faults and various sensor fault detection mechanisms of existing works; Sect. 3 presents the proposed methodology; Sect. 4 provides the findings and discussion of the proposed work; and Sect. 5 concludes with recommendations for further research.

## Related works

Many researchers have proposed various methods for detecting the sensor node failures and providing efficient localization among them. Based on existing approaches, this section is divided into three subsections, namely, fuzzy-based, self diagnosis-based, and machine learning, deep learning and optimization based fault detection approaches.

### Fuzzy-based Fault Detection Approaches

In WSN, the authors suggested an ANFIS, a decentralized faulty node detection and categorization approach^20^. The proposed scheme categorizes sensor node faults based on the sensor nodes’ crisper performance measure. The nodes in this system are classified as healthy, lifeless, dispatch, resting, or end according to their crisper performance measure. This ANFIS scheme detects the sensor node faults by intra-cluster and inter-cluster failure detection estimators. The limitations are that security elements in this work are not addressed in this system. In another approach, for heterogeneous WSNs, the authors^21^ introduced a distributed fuzzy logic-based faulty node identification technique. This system provides a weighted voting mechanism that uses fuzzy logic in order to recognize the various sensor node faults and occurrences in the deployed area. This self-diagnosis system uses an approach based on spatial correlation to pinpoint the SN failure in the deployed region. Every SN in the monitoring region is assigned a weight according to the fuzzy logic controller it has. The controller treats a node as faulty if it detects weighted values that differ from those of its surrounding nodes. Moreover, according to the sensed value, location, distance and coverage parameters, this technique is used to discover and restore the faulty sensor node. The main disadvantage of this system is that it completely depends on its neighbor sensor nodes, and it reduces sensor nodes fault detection accuracy.

For WSNs, a FNCM approach based on fuzzy rules is presented in^22^ with the goal of detecting and reusing faulty nodes. The faulty sensor nodes are correctly reused during the data routing procedure using that method. The data routing technique increases the usability of malfunctioning nodes and optimizing network lifetime. The estimation of energy gain is improved by reusing a faulty node from the proposed work. Different nodes are categorized using a fuzzy interference model in accordance with specified functions and the defuzzifier system generates a function to obtain the distinct node faults. This system can be utilized for road monitoring, home automation and livestock management in order to identify physical or environmental situations. Moreover, it improves the usability of restored faulty nodes and uses fuzzy logic to get around WSN’s uncertainty. The drawbacks include the difficulty for a fuzzy inference model to create prior knowledge about the node condition because of the inherent self-organization feature.

### Self-diagnosis approaches

An intelligent AI-driven hyperparameter-tuned LSVM fault diagnosis approach is proposed by Kaur and Bhattacharya^23^ to improve the reliability and longevity of sensor networks. In order to achieve a hassle-free communication path design, their approach employs the reinforcement learning technique. Moreover, their approach offers self-learning capability to the sink, which greatly extends the network lifespan and grants the system autonomy.

The author suggested FMMC technique can identify faulty sensor nodes and classify sensor faults based on their hardware sensor faults^24^ in WSN. The Markov method makes it possible to assess node state and improve fault detection precision. In comparison to other methods, this model uses self-detection to evaluate node results, which consumes less energy. The FMMC model fixes all persistent and intermittent faults, restores the faulty SNs, and assigns updated states for faulty SNs depending on their current hardware state. The disadvantages of this approach include the inability to detect faults in network connectivity perception.

For dynamically determining the performance of live SNs, fixing faulty nodes, and locating the best alternate routing solution, the authors’ proposed OEPO technique^25^. In their model, by analyzing the fitness function for each SN, the faulty nodes are located using the cluster boundary value and the fitness value. This model enhances the performance and permanence of the network during the self-diagnosis process. Due to the fitness function that is suggested in this model, the detection accuracy produced by this model is lower.

### Machine learning, deep learning and optimization based approaches

The Harmony Search Algorithm (HSA)^4^ is used to determine which nodes are defective in WSN. In order to detect the faulty sensor nodes in WSN, HSA bases its algorithms on meta-heuristics and employs a probabilistic search-focused method. The correlation and energy values of neighboring nodes are included in each memory vector of the HSA for identifying the sensor node problems. The advantages are better node fault detection produces. One of the limitations of their work is that estimating correlation could result in increased processing overhead.

A faulty node detection technique was proposed by Nagaraja and Mahadevaswamy^26^ to ensure effective data transmission in sensor networks. Their method isolates the faulty nodes within the network and, identifies and diagnoses transmission path faults. However, the computational overhead and complexity associated with employing their technique to identify faulty SNs impose limitations on their research.

Thiyagarajan et al^27^. proposed a fault detection and data recovery approach by utilizing FDSCAN approach, which identifies and isolates faulty nodes within the network. In their approach, Bi-LSTM is employed to address and recover the missing data, ensuring the data integrity. However, their research is constrained by the computational overhead and complexity involved in accurately pinpointing the faulty SNs in the network.

The authors^28^ proposed a feed-forward neural network based approach called GSPSO. It is an automated fault node identification technique that makes use of feed-forward neural networks that were hybrid and meta-heuristic trained. This proposed model is not suitable for transition fault detection because the transition fault state was unable to be captured by neural networks on its own. This method analyses both sensor and communication connection issues in addition to sensor faults. The limitations are that only homogeneous nodes can be used with this method.

For WSN, the authors of^29^ proposed a distributed CLAFNDM strategy for identifying faulty sensor nodes based on hardware condition and reusing those faulty nodes. The status of each SN is computed by cellular learning automata based on the sensor node’s hardware configuration. For efficient identification of the faults, CLA employs eight rules and a suitable machine learning methodology is employed. These eight guidelines were created so that, in the event that CLA makes incorrect decision, it can be corrected in the subsequent round and the correct outcome can be confirmed.

Palani et al^30^. presented a method for detecting and recovering from hard faults in sensor nodes. Their approach integrates deep-reinforcement learning (DRL) with the hosted cuckoo optimization algorithm to identify and recover hardware faults in sensor networks. This technique aims to extend the network’s lifetime while minimizing energy consumption and enhancing the accuracy and quality of large-scale WSNs. However, the method has limitations; it is not suitable for diagnosing persistent WSN issues and is only applicable to intermittent sensor SN faults.

A hybrid method has been presented by Gouda et al^31^. to diagnose intermittent SN faults in WSN. They employed the likelihood ratio test as part of their methodology to identify and diagnose the state of faulty SNs that exist in WSN. Additionally, they have utilized an algorithm in their methodology that senses changes in variance and mean to diagnose intermittent faults. A key feature of their approach is its applicability to both centralized and distributed systems. Their methodology’s shortcomings include its limited effectiveness for random and spike faults in SNs and its inappropriateness for diagnosing other kinds of WSN faults. In another hybrid approach, Fan et al^32^. integrate machine learning with evolutionary computing to identify and diagnose faults in WSN. Their approach facilitates precise identification of invalid data and ensures the proper function of WSN. However, their work has limitations, as it utilizes advanced mobile sinks to collect data and can negatively impact network performance. Table 4 presents the comprehensive summary of existing approaches.

**Table 2:** Comprehensive summary of existing approaches.

Author and Year	Methodology	Strengths	Limitations	Fault Detection Category	Fault Recovery
Rajan et al., 2021 [11]	Adaptive Neuro-Fuzzy Inference System	To detect and isolate the faulty clusters and SNs from network data collection process	Security elements in this work are not addressed	Fuzzy based fault detection / Distributed	No
Masdari et al., 2020 [12]	Weighted voting mechanism with fuzzy logic	The capability to identify transitory faults more precisely while reducing the occurrence of false positives	Completely depends on its neighbor sensor nodes, and it reduces sensor nodes fault detection accuracy	Fuzzy based fault detection / Distributed	No
Chanak & Banerjee. 2016 [13]	Faulty-node classification & management (FNCM)	Improves the usability of restored faulty nodes	difficulty for a fuzzy inference model to create prior knowledge about the node condition	Fuzzy based Fault detection / Distributed	Yes
Nagaraja & Mahadevaswamy, 2022 [40]	Adaptive Zigbee-Aquila communication protocol (AZACP)	Focuses on identifying faults and data transfer delays	Computational overhead	Distributed	No
Moridi et al., 2020 [16]	FMMC	Both permanent and transient faults are addressed	Inability to detect faults in network connectivity perception	Self-detection / Distributed	Yes
Kumar and Rao, 2021 [20]	OEPO	Enhances the performance and permanence of the network	Lower detection accuracy	Self-diagnosis / Distributed	No
Kaur & Bhattacharya, 2023 [15]	AI-driven hyperparameter-tuned LSVM	Extends the network lifespan and grants the system autonomy	Not tested in dynamic environments	Self-learning	No
Mosavvar & Ghaffari, 2018 [4]	Harmony Search Algorithm	Better fault detection	Estimating correlation could result in increased processing overhead	Probability based fault detection / Distributed	No
Thiyagarajan et al., 2024 [17]	FDSCAN and Bi-LSTM networks	Improved accuracy and network reliability	Not tested in dynamic environments	Deep Learning	Yes
Swain et al., 2020 [18]	GSPSO	Analyze sensor faults as well as communication path fault	Only homogeneous nodes can be used	Neural Network	No
Gouda et al., 2023 [19]	Likelihood ratio test	It can apply to both centralized and distributed systems	Limited effectiveness for random and spike faults	Hybrid (both centralized and distributed)	Yes
Fan et al., 2023 [21]	Machine leaning with evolutionary computing, BPNN	Precise identification of Fault data	Computational overhead	Machine Learning	No
Palani et al., 2022 [34]	DRL and hosted cuckoo optimization	Extends the network’s lifetime while minimizing energy consumption and enhancing the accuracy and quality of large-scale WSN	Not suitable for diagnosing persistent WSN issues	Distributed	Yes

### Research Gaps

The studies on handling faults in WSNs described earlier have pointed out several shortcomings. Due to the resource-constrained nature of SNs, maintaining the stability of sensor networks with faults has proven challenging for most existing solutions. The primary concern is that most of the existing approaches are unable to address diverse faults, making it difficult to manage multiple types of sensor faults simultaneously. Recent advancements have focused on collaborative techniques for identifying, diagnosing and classifying different types of sensor faults. However, these approaches frequently encounter hardware incompatibility issues as the hardware of the SN is not compatible with existing fault diagnosis methods. Due to the substantial computational overhead during fault diagnosis, particularly as network size and fault probability increase, further straining these limited resources.

To tackle these challenges, the proposed protocol employs efficient fault detection, diagnosis and classification algorithms based on PHMM and FCSO. Moreover, the proposed protocol uses the FCSO to locate the faulty SNs and the restoration algorithm employed to isolate and recover the faulty SNs in the network. Table 5 depicts the notations are utilized in this proposed protocol and their meaning.

**Table 3:** Notations.

Notations	Meaning
n_i_	Sensor node
SN_i_	Unique identifier of sensor node
$\:\text{a}{n}_{i},\:\text{b}{n}_{i}$	Coordinates
NN(ni)	Neighbour node
R_t_	Range of transmission
$\:\text{d}\text{e}\text{g}\left({n}_{i}\right)$	Degree of sensor node
$\:\alpha\:$	Node count
$\:{P}_{r}$	Received signal strength
Σ	Wavelength
P_r_, P_t_	Received and transmitted power
G_r_, G_t_	received and transmitted gains
t_out_	Time frame
T_out_	Timout period
TH_p_, TH_i_, TH_t_	Threshold values for permanent, intermittent and transient faults
ACK	Acknowledgement
Θp, Θi, Θt	Predefined threshold values
Stat_i_	Status of node
mean_t_	Mean of each sensor node
ε_t_	Residual energy
X_ijt_	Position of chicken
CN	Number of chicken
$\:{y}_{i,j}^{t+1}$	Location of rooster
k	Maximum of rooster
H_1_, H_2_	Learning factor
f_i_, f_k_	Fitness values
rand, naggr	Random values
MG	Maximum generation

## Proposed Sensor Node Fault Detection Scheme

**Fig. 1:**
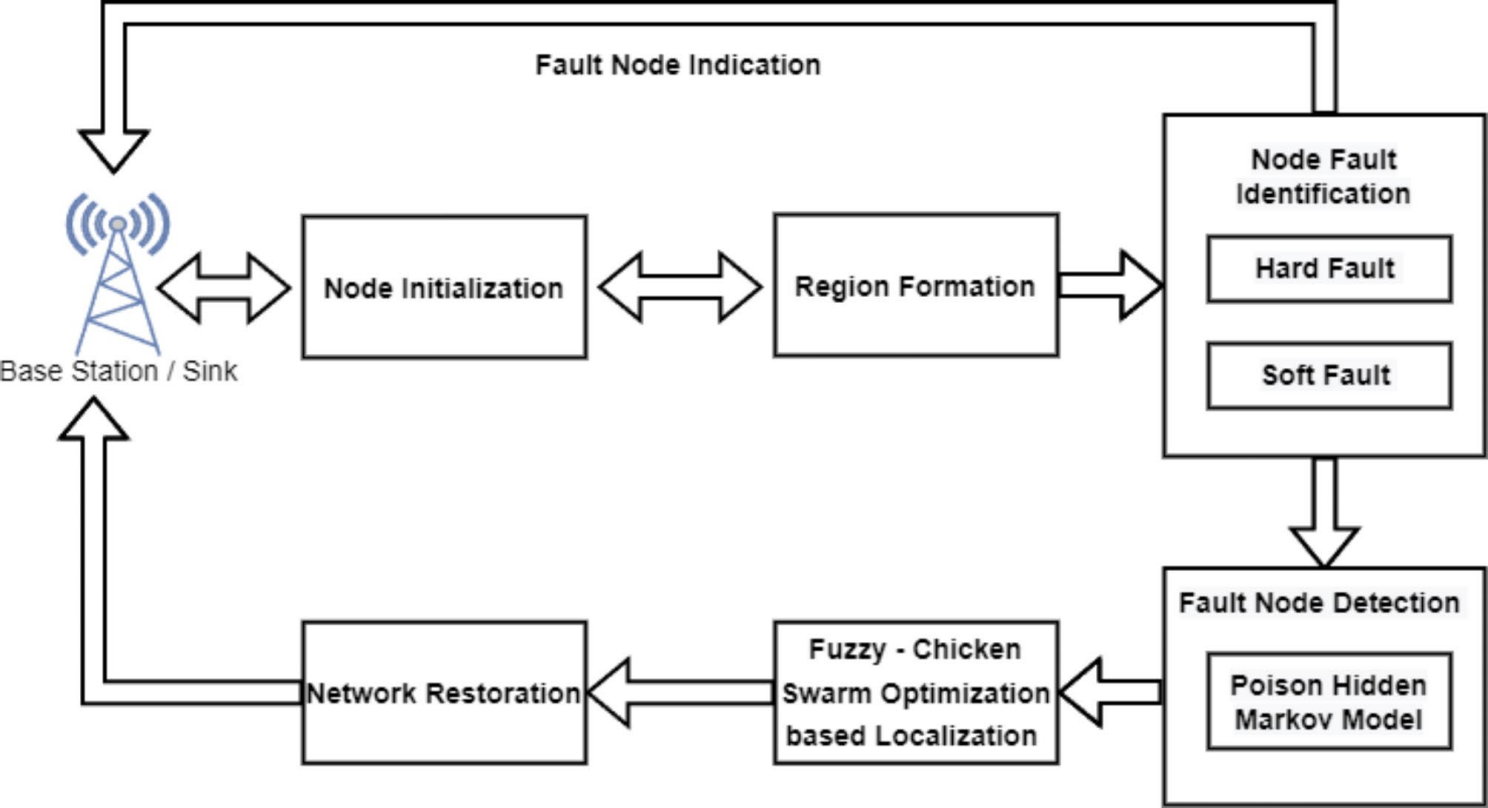
Architecture of proposed system.

Figure 1 represents the proposed PHMM-FCSO architecture. The proposed system consists of five major modules namely, node initialization and region formation phase, sensor node fault diagnosis phase, sensor node fault identification phase, faulty sensor node localization phase and faulty sensor node restoration phase. The main aim of the node initialization and region formation phase is to initialize the nodes and form the region among the nodes. The next module is the sensor node fault identification phase. The main aim of this phase is to diagnose hardware sensor node fault detection and software node fault detection. The next module is the faulty sensor node detection phase. The primary goal of this phase is to discover and identify faulty sensor nodes using the Poisson Hidden Markov Model (PHMM). The next phase is the localization phase. The primary goal of this phase is to locate the faulty SNs by employing fuzzy-based chicken optimization (F-CSO) algorithm. The next phase is the faulty sensor node restoration phase. The primary purpose of this network restoration phase is to remove and restore the faulty sensor nodes in the given region of WSN.

The proposed PHMM and F-CSO algorithms are implemented at the base station (BS), leveraging its superior computational power, battery, and memory to reduce the burden on the SNs. Utilizing realistic parameters, the proposed algorithms detect soft and hard faults and relay the fault information to the BS. The BS then determines the location of the faulty SNs within the network and initiates the recovery process. If the recovery process is successful in case of transient or intermittent faults, the identified faulty SNs are restored to the network. In contrast, if a permanent fault is detected, the BS isolates the faulty SNs and broadcasts the fault information to the network administrator.

### Node initialization and region formation phase

In the proposed system, let us consider the network with sensor nodes SN such that M= {n_i_: 1 ≤ i ≤ SN}. Each network node is independent and deployed at random over a Z x Z square unit area^33^. Every sensor node (n_i_) has a distinct identifier (SNi), is deployed in a location that may be inaccessible to humans, has coordinates (an_i,_ bn_i_), and it satisfies the Eq. (1).

1 $$\:0\le\:\text{a}{n}_{i},\:\text{b}{n}_{i}\le\:\text{Z}$$

Each sensor node in the 1-hop communication range, designated as n_i_, communicates with its neighbouring nodes NN(ni). R_t_ is range of transmission, homogeneous SNs is larger than or equal to the Euclidean distance of two nodes, n_i_ and n_j_, is larger than or equal to the transmission range R_t_ of homogeneous sensor nodes. The Eq. (2) gives the representation of the Euclidean distance of between n_i_ and n_j_ nodes:

2 $$\:{d(n}_{i},\:{n}_{\text{j}})=\sqrt{{(\text{a}{n}_{i}-\text{a}{n}_{\text{j}})}^{2}+{(\text{b}{n}_{i}-\text{b}{n}_{\text{j}})}^{2}}\:\le\:\:{T}_{r}$$

The MAC protocol is used by sensor nodes in the proposed IEEE 802.15.4 standard to establish connections with one-hop neighbours that are located within the transmission range. The number of SNs within the range of transmission determines a node’s degree (i.e., one-hop neighbour sensor nodes). The degree of sensor nodes is calculated by Eqs. (3) and (4):

3 $$\:\text{d}\text{e}\text{g}\left({n}_{i}\right)=\sum\:_{\text{k}=1}^{\text{N}\text{N}\left({n}_{i}\right)}{n}_{j}$$

where

4 $$\:{d(n}_{i},\:{n}_{\text{j}})\:\le\:\:{R}_{t}$$

Once the sensor node initialization is completed, the next step is the region formation phase. In the proposed system, the region formation plays a major role in achieving the energy gain with fault detection accuracy. To form the network into R regions, the network is divided into several separate regions with n nodes. The average node count α in the region is calculated using Eq. (5).

5 $$\:\alpha\:=n/R$$

The ratio between a residual energy and its average of sensor nodes determines which nodes in the network are chosen to be members of each non-overlapping region in the network. The formation of a region depends on the energy efficiency between the region members and the region head^34^. The high-energy node is elected as the region head and there are no isolated nodes in the network. The region head is elected by employing received signal strength (P_r_), and that is calculated by using Eq. (6).

6 $$\:{P}_{r}=\:{P}_{t}{G}_{t}{G}_{r}{\left(\frac{{\upsigma\:}}{4{\uppi\:}\text{d}}\right)}^{2}$$

where σ is the wavelength, P_r_ and P_t_ are the received and transmitted powers, G_r_ and G_t_ are the received and transmitted gains, and distance between the receiver and sender nodes is denoted as d. The notation denotes the division of the entire network into various regions based on the values of R, α, and RSS and is represented by the notation R={R_1_,R_2_,…,R_x_}.

### Sensor Node Fault diagnosis phase

The next step in the proposed system is the SN fault diagnosis phase. The main objective of this phase is to diagnose and identify the faults of SNs deployed in the respective regions. In the proposed system, the sensor node fault diagnosis is subdivided into two major phases, namely, hard and soft sensor node fault detection phases^35,36^. In the hard fault detection phase, the faults related to hardware components of the sensor are identified. In the soft fault detection phase, the faults related to the software components of the sensor nodes are identified.

#### Hard Sensor Fault Detection Phase

This algorithm identifies the occurrence of hardware-based faults in the nodes of the network. The status of every SN in the region is maintained by every sink or base station. The variable Stat_ij_ is used to represent the status of sensor nodes. The base station transmits a fresh message in the entire region at each t time duration. Each member node sends an acknowledgement (ACK) to the base station after receipt a message, the ACK must be delivered within the specified time frame, t_out_. The SN is announced as faulty and its status is set to 1 if the ACK is not received for the predetermined amount of time. When a node experiences a transient fault, its status is changed to 1 if the time period t_out_ exceeds the time T_t_. When a node experiences an intermittent fault, its status is set to 1 if the time period t_out_ exceeds the time T_i_. When a node experiences a permanent fault, its status is changed to 1 if the time period t_out_ exceeds the time T_p_. The SN is announced as hard sensor fault, when$\sum\limits_{{i=1}}^{{NN(nk)}} {{S_{ik}}} \geqslant \left[ {\frac{{NN(nk)}}{2}} \right]$ is true. Then the status of each node can also be determined similarly. Algorithm 1 describes the hard fault detection process.

**Fig. a:**
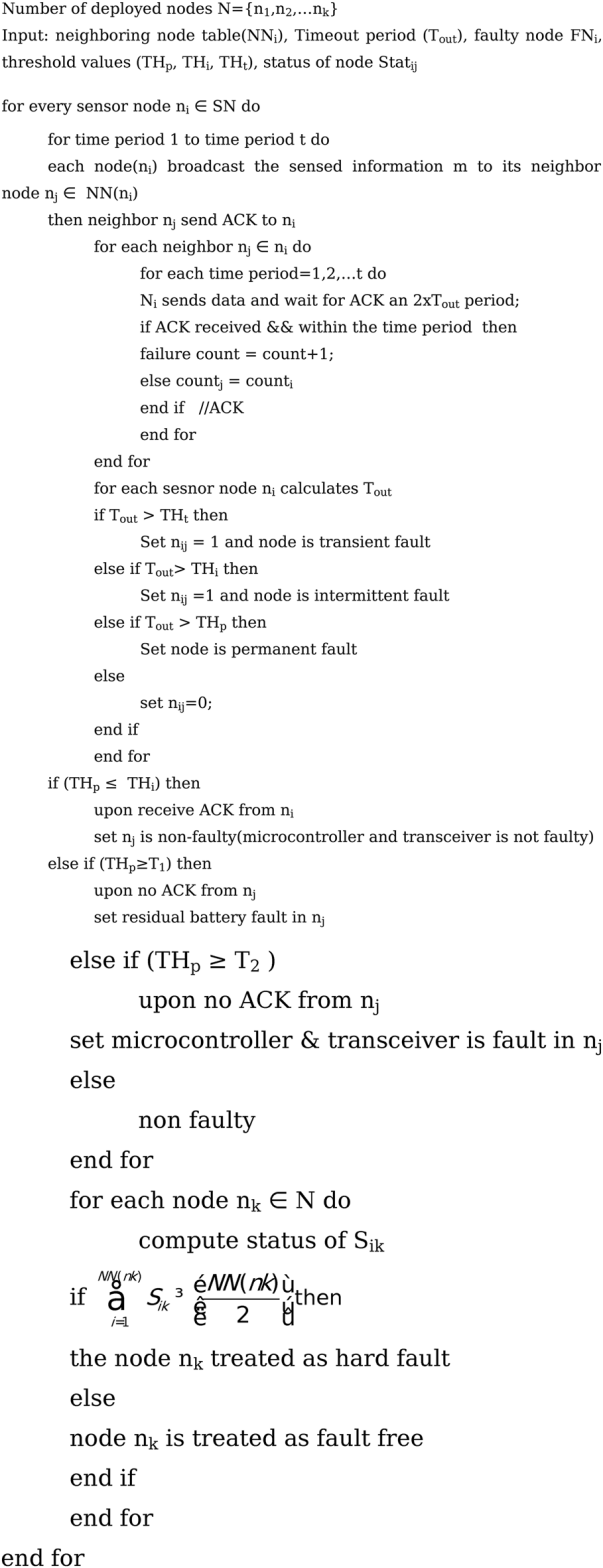
Algorithm

#### Soft Sensor Fault Detection phase

Once the hard fault phase is completed, the next step is to detect the faults based on software. The soft faults in the WSN are grouped as permanent, intermittent and transient soft fault^15,37^. Initially, each SN n_i_ broadcasts its sensed sensor information to the sink or base station in the communication range of a region. Then the base station detects and identifies the presence of faulty sensor nodes in the region by comparing sensed data with the predefined threshold values Θp, Θi, Θt to identify the type of faults. The fault status of each node can also be determined similarly. Algorithm 2 gives the steps involved in the soft fault detection phase.

**Fig. b:**
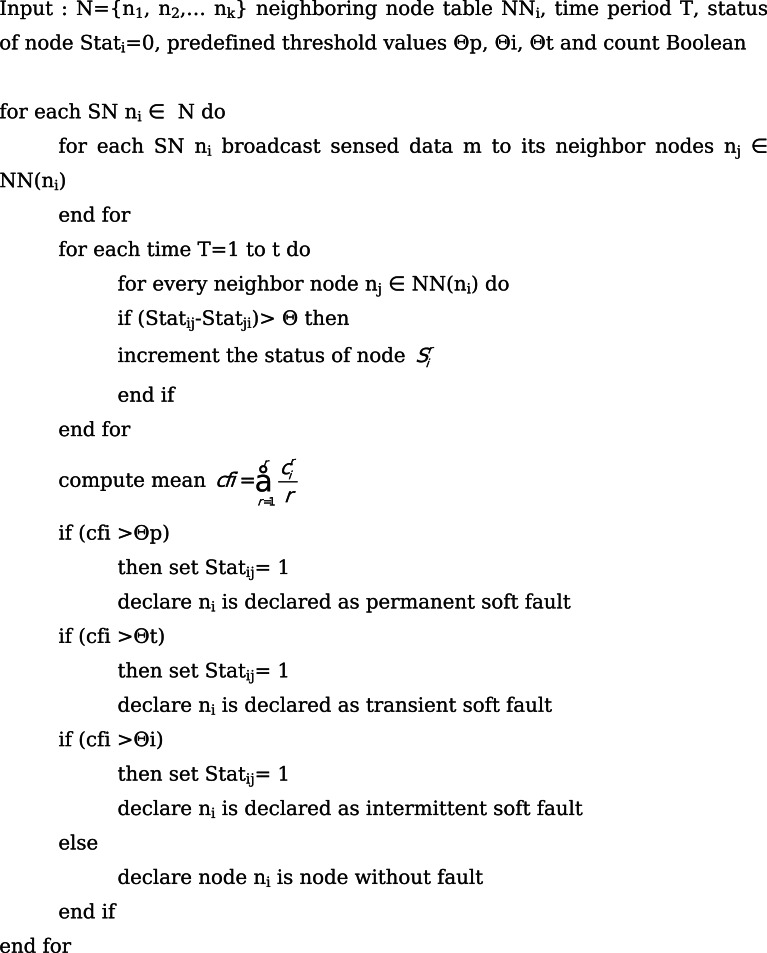
Algorithm

### Sensor Node Fault Identification Phase

The next phase of the proposed system is the sensor node fault identification stage. The primary purpose of this stage is to detect and identify the sensor hard fault and sensor soft fault present in WSN. The network’s nodes are denoted as {n_1_,n_2_,…,n_k_}, where k indicates the maximum number of SNs in the network. The regions formed from that network are written as R={R_1_,R_2_,…,R_x_}, where x is the number of regions formed. The damaged region of the network is determined by using the Poisson distribution. Each region and its nodes can be denoted as R_x_={n_ij_,…,n_qm_}, where qm indicates the region number and total number of nodes in that particular region.

In the proposed system, Poisson distribution is used to determine the faulty nodes in the region by using the standard deviation and mean of each node. The mean of a particular node in the given region is calculated from the sensed data of each neighborhood sensor node and the standard deviation of sensed data. The mean of a particular node in the given region is calculated using Eq. (7).

Mean_i_=

7 $$\frac{1}{{Ri}}\sum\limits_{{j\epsilon Ri/Ni}}^{{}} {{M_j}}$$

Standard Deviation of the nodes in the particular region is calculated by using Eq. (8).

SD_i_ =

8 $$\frac{1}{{Ri - 1}}\sum\limits_{{j \epsilon Ri/Ni}}^{{}} {{M_j} - Mea{n_i}}$$

The each region‘s mean is computed by using Eq. (9).

Mean =

9 $$\frac{1}{q}\sum\limits_{{j \epsilon{R_i}/{N_i}}}^{{}} {Mea{n_i}}$$

#### Faulty Node detection using Poisson Hidden Markov Model (PHMM)

The proposed fault detection system Poisson Hidden Markov Model (PHMM)^38^ is employed for detecting the faulty sensor nodes by computing the active of nodes using the Poisson distribution. The model equation for Poisson distribution for the nodes is defined by the Eq. (10)

10 $${y_t}=mea{n_{t}}+{\epsilon _t}$$

where mean_t_ is the sum of mean calculated by the observed value of each sensor and ε_t_ is the residual error and it is constant variance which is normally distributed as random variables. The probability mass function of y is computed by using Eq. (11)

11 $$P\left( {y={y_t}|Mea{n_t}} \right)=\frac{{{e^{Mea{n_t}}}Mea{n_t}_{{}}^{{{y_t}}}}}{{{y_t}!}}$$

where probability of Poisson distributed y with Mean_t_.

Considering k state Markov process that is to be assumed in some state j∈[1,2,3,… k] which is shown in Eq. (12) when s_t_ = j 

12 $${y_t}=mea{n_{tj}}+{\epsilon _t}$$

The exponentiated mean of Poisson Hidden Markov model is computed by using Eq. (13)

13 $$Mea{n_{tj}}=e_{{}}^{{xt}}{b_t}$$

The probability predicted Hidden Markov model of observing y_t_ at time t is computed by using Eq. 

14 $$P\left( {y={y_t}|Mea{n_t}} \right)=\sum\limits_{{j=1}}^{k} {\left[ {\left( {\frac{{{e^{Mea{n_{tj}}}}Mean_{{tj}}^{{{y_t}}}}}{{{y_t}!}}} \right)P\left( {{s_t}=j} \right)} \right]}$$

The Poisson Hidden Markov model has been used to detect the both software and hardware sensor faults in the cluster region of WSN. The proposed technique accepts input from various original datasets, returns the fault-injected dataset, and then passes the dataset to determine the accuracy of the fault node detection. The proposed Poisson Hidden Markov Model fault detection algorithm is described in algorithm 3.

**Fig. c:**
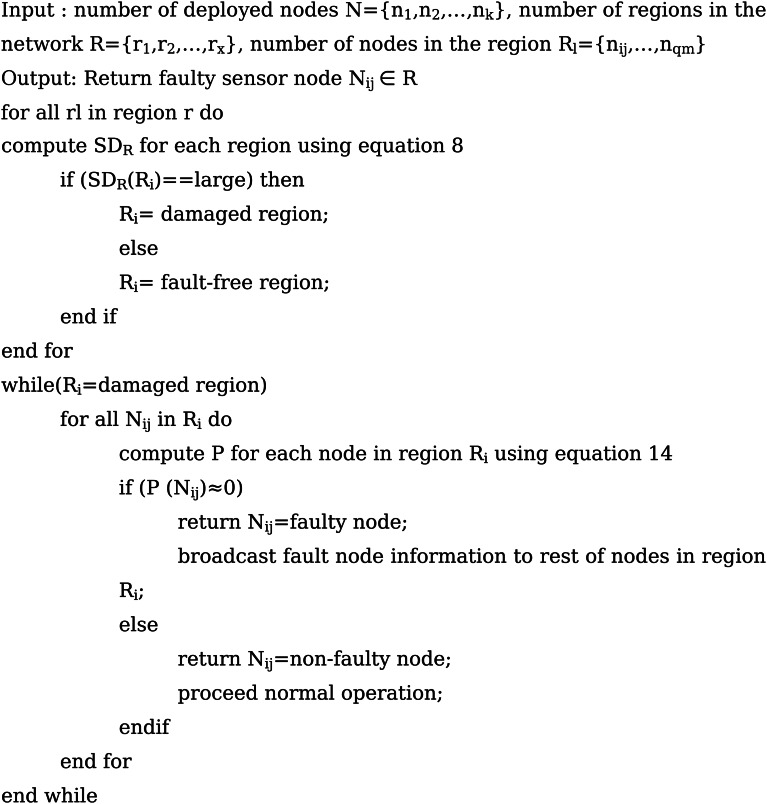
Algorithm

### Faulty Sensor Node localization phase

The next phase is the faulty sensor node localization phase. The main objective of the faulty sensor node localization phase is to identify and localize^39,40^ the faulty sensor node from the given region. To identify and locate the faulty sensor node from the given region, the proposed system employs a fuzzy-based chicken swarm optimization algorithm. The location of the faulty sensor node is determined by geographic representation of the network. The fuzzy-based Chicken Swarm Optimization (F-CSO) provides a precise and efficient operation to identify the exact location of the faults.

#### Fuzzy based Chicken Swarm Optimization (F-CSO)

The bio-inspired chicken swarm optimization (CSO) algorithm^41,42^ is an advanced intelligent algorithm which represents the different behaviors related to chickens, hens and cocks in their process of food search. It is a random search algorithm that resembles the hierarchical structure and behaviour of the chicken swarm. The CSO approach has many subgroups, each subgroup in CSO, consists of several chicks, few hens and a rooster. The rooster has the highest fitness value and rooster act as leaders of each subgroups. The chicks have worst fitness values and it is randomly grouped into subgroup. The positions of each rooster, hens, and chicks represent solution to a problem.

The identities of the rooster, hens and chicks are determined by their fitness value (energy) which is calculated by Eq. 16. The nodes with the highest fitness value are called roosters. The rooster numbers indicate the maximum number of subgroups in the network. The remaining nodes are referred to as hens, while the nodes with the least fitness value are referred to as chicks. In CSO, few numbers of hens and several chicks are randomly formed as a subgroup. To establish relationships with the chicks, the predetermined number of hens is picked at random intervals.

**Fig. 2:**
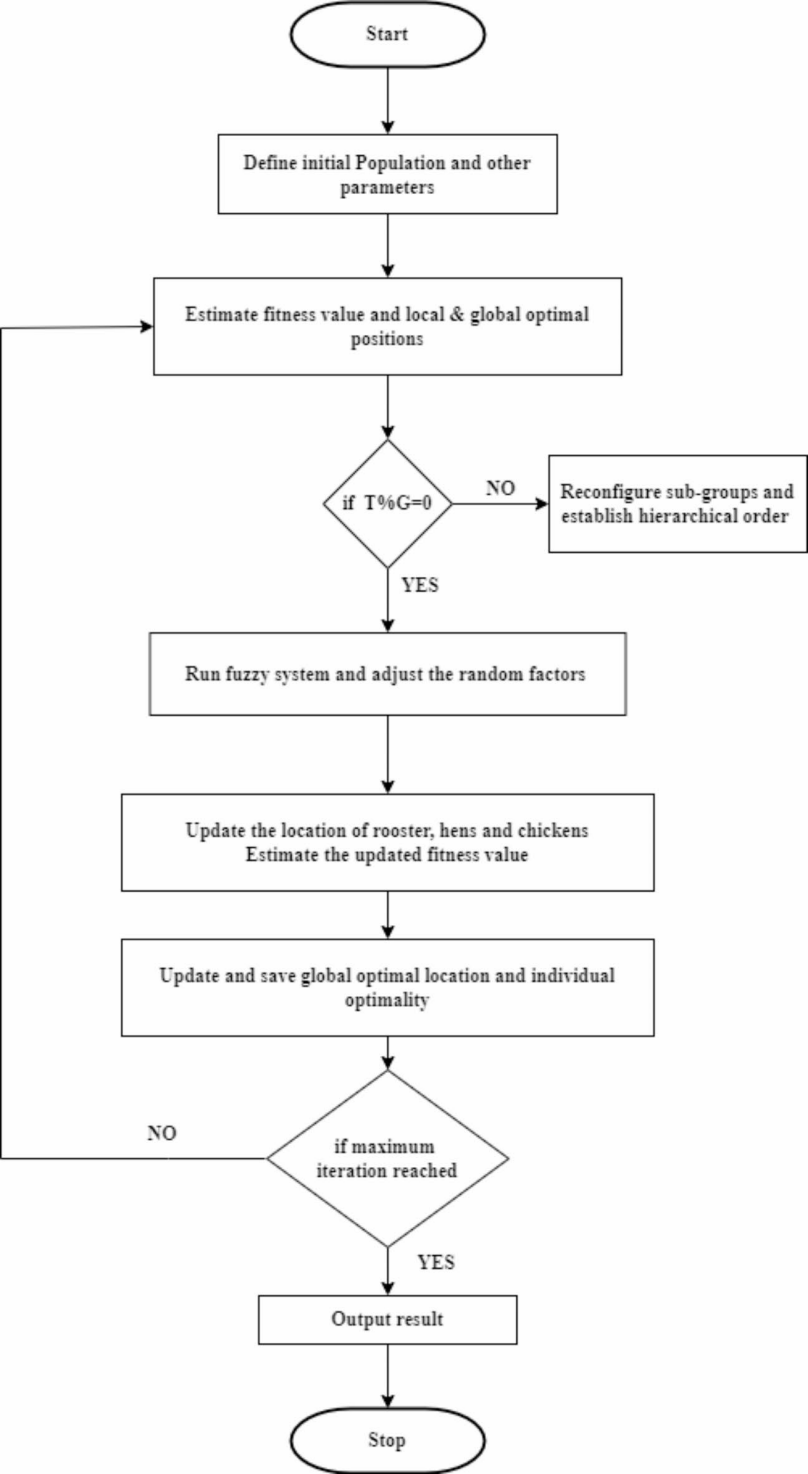
Stages of F-CSO.

The Fuzzy-based CSO (F-CSO) algorithm is proposed to identify the faulty sensor node’s location. The random factors rand and F are completely random, at the initial stage of iteration (the first few iterations), we would have a large searching space in order to identify the global optimum as much as feasible. The general steps of F-CSO are shown in Fig. 2. Based on Eqs. 22 and 23, the random factors rand and naggr have a large searching range, which map the fuzzy values high, very high, and medium accordingly. When the F-CSO algorithm would have narrowing random factors of medium, very low, and low while running on its final stage of iterations (large iteration). When the optimization speed and iteration time are medium, we have mapped the fuzzy values as high, medium and low.

The position of each chicken is denoted by X_ijt_ and it is obtained by calculating at the with t^th^ iteration, i^th^ chicken and the j^th^ dimension, then iε{1,…,CN}, jε{1,…,DN}, tε{1,…,IN}, where CN indicates the number of chickens (nodes), DN stands for the dimension number and IN represents iteration.

Rooster position.

The location of the rooster is evaluated by using the Eq. (15)

15 $$\:{y}_{i,j}^{t+1}=\:{y}_{i,j}^{t}*(1+)\Phi (0,\sigma _{{}}^{2})$$

where

16 $$\sigma _{{}}^{2}=\left\{ {\begin{array}{*{20}{c}} {1,} \\ {\exp \left( {\frac{{{f_k} - {f_i}}}{{|{f_i}|+\begin{array}{*{20}{l}} {\mathbf{\varepsilon }} \end{array}}}} \right),} \end{array}\begin{array}{*{20}{c}} {} \\ {k \epsilon [1,RN],k \ne i} \end{array}} \right\}$$

where k is the maximum of roosters chosen at random, f_i_ and f_k_ are the ith and kth roosters fitness values of the, ε is a constant and $\Phi (0,\sigma _{{}}^{2})$ is a random number derived by Gaussian distribution function with an expectation of zero and variance ($\sigma _{{}}^{2}$).

Hen position.

The position of hen is determined by using the Eq. (17)

17 $$\:{y}_{i,j}={y}_{i,j}\left(t\right)+{H}_{1}.rand.({y}_{r1,j}\left(t\right)-{y}_{i,j}\left(t\right)+{H}_{2}.rand.({y}_{r2,j}\left(t\right)-{y}_{i,j}\left(t\right))$$

Here H_1_ and H_2_ are the learning factors, r_1_ is the rooster index, r_2_ is number of a rooster or hen that is chosen at random, and r_1≠_ r_2_. Here H_1_, H_2_ is computed by using the Eqs. (18) and (19)

18 $$\:{H}_{1}=\text{e}\text{x}\text{p}\left(\frac{{f}_{ih}-{f}_{r1}}{abs\left({f}_{i}+\epsilon\right)}\right)$$

19 $$\:{H}_{2}=\text{e}\text{x}\text{p}({f}_{r2}-{f}_{i})$$

Chicken position.

The position of chick is calculated by using the Eq. (20)

20 $$\:{y}_{i,j}(t+1)={y}_{i,j}\left(t\right)+F.({y}_{m,j}\left(t\right)-{y}_{i,j}\left(t\right))$$

Here F is a random factor with a range between 0 and 2 and y_m, j_ (t) is the mother hen of chicks.

IF-THEN fuzzy rules are formulated using the fuzzy proposition. The random factors values can be adjusted by using the following set of rules

21 $$R_{n}^{{(r)}} = if < F_{1}^{r}> then < F_{2}^{r}> ,r = 1,2, \ldots ,9$$

The random rand, naggr value are computed by using the Eq. 

22 $$rand=\left\{ {\begin{array}{*{20}{c}} {\frac{1}{{2\prod *\alpha }}\exp \left( { - \frac{{ran{d^2}}}{{2\sigma _{{}}^{2}}}} \right),rand \leqslant 1} \\ {0,otherwise} \end{array}} \right\}$$

23 $$naggr=\left\{ {\begin{array}{*{20}{c}} {\frac{1}{{2\prod *\alpha }}\exp \left( { - \frac{{nagg{r^2}}}{{2\sigma _{{}}^{2}}}} \right),naggr \leqslant 1} \\ {0,otherwise} \end{array}} \right\}$$

**Fig. d:**
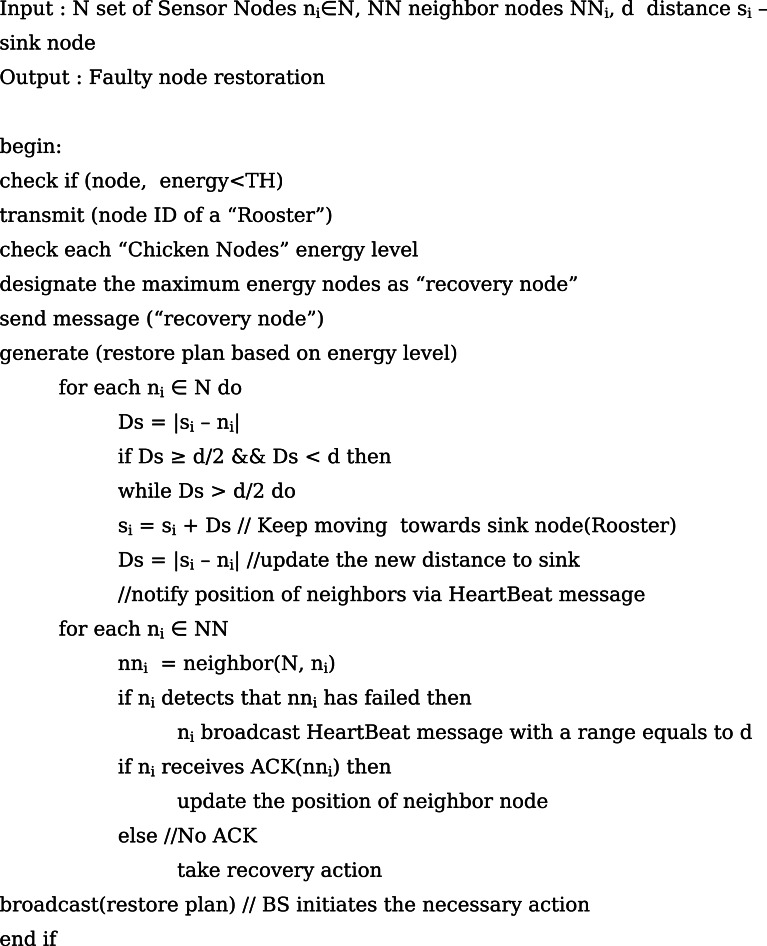
Algorithm

#### Fuzzy rule mapping for adjusting chickens

Table 4 displays the fuzzy rules mapping for modifying the number of chickens (nodes) used by the fuzzy inference system. The fuzzy inference systems take two parameters optimization speed and chicken aggregation to adjust the number of subgruop member nodes (or “chicks”) in the network.

**Table 4:** Fuzzy rule mapping for adjusting chickens.

S.no	Fuzzy rules for adjusting chickens
**1**	IF (Optimization speed is Low) and (Chicken Aggregation is Low) THEN (number of chicken is Medium possible)
**2**	IF (Optimization speed is Low) and (Chicken Aggregation is Medium) THEN (number of chicken is High possible)
**3**	IF (Optimization speed is Low) and (Chicken Aggregation is High) THEN (number of chicken is Very High possible)
**4**	IF (Optimization speed is Medium) and (Chicken Aggregation is Low) THEN (number of chicken is Low possible)
**5**	IF (Optimization speed is Medium) and (Chicken Aggregation is Medium) THEN (number of chicken is Medium possible)
**6**	IF (Optimization speed is Medium) and (Chicken Aggregation is High) THEN (number of chicken is High possible)
**7**	IF (Optimization speed is High) and (Chicken Aggregation is Low) THEN (number of chicken is Very Low possible)
**8**	IF (Optimization speed is High) and (Chicken Aggregation is Medium) THEN (number of chicken is Low possible)
**9**	IF (Optimization speed is High) and (Chicken Aggregation is High) THEN (number of chicken is Very Medium possible)

### Faulty Sensor Node Restoration

The next phase is the faulty sensor node restoration approach demonstrating the node relocation procedures. Each node in the network will broadcast hello messages with a transmission range to inform other nodes of its position. In order to acknowledge one another, each node exchanges data such as its ID and current position (ACK). Moreover, every node in the network transmits the sensed data within the broadcasting range on occasion. This broadcast message is used to identify the failing nodes in that region. If there is no broadcast message, the presence of the indication of failure nodes is assumed. By doing so, each node refreshes its list of neighbours and initiates the network’s mobility process. The BS sends out notifications to network managers, providing comprehensive details about the types of faults. It also initiates the necessary actions to recover from transient or intermittent faults and to restore faulty nodes when the SNs fail. Algorithm 5 gives the steps involved in the faulty sensor restoration phase.

**Fig. e:**
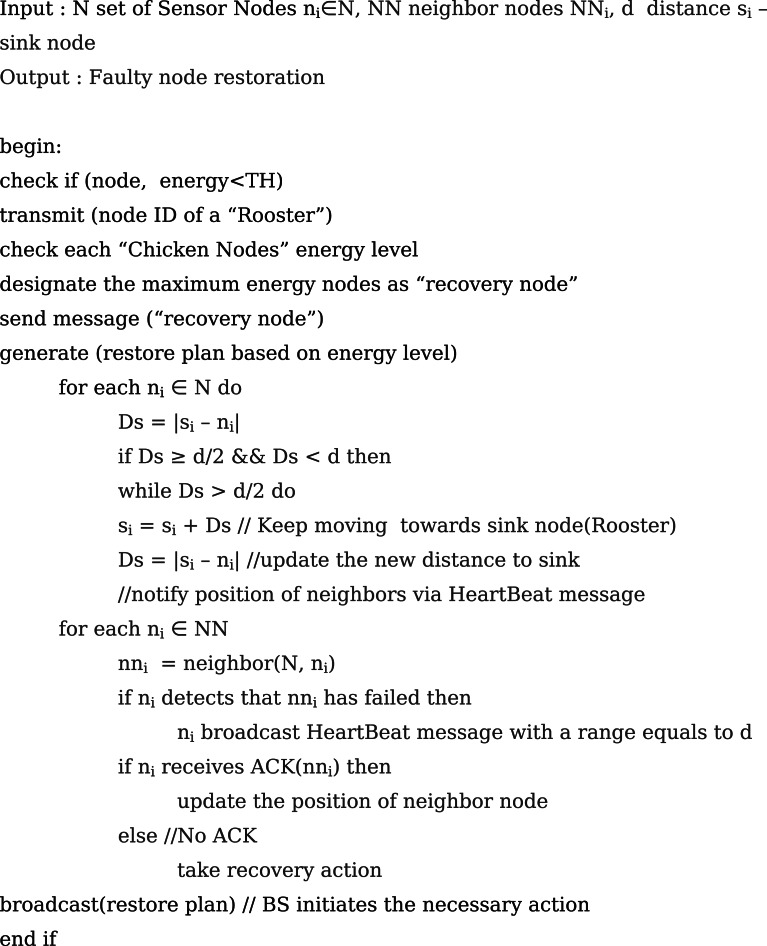
Algorithm

## Results and discussion

The network simulator NS2 is used to implement the proposed system Poisson Hidden Markov Model (PHMM) and fuzzy rule based chicken swarm optimization (F-CSO) algorithm under the Windows environment. On a wireless sensor network, the proposed PHMM-FCSO model is evaluated using the parameters enlisted in Table 5. The effectiveness of the proposed system (PHMM-FCSO) is compared with four existing models BPNN^32^, OEPO^25^, DRL^30^and HAS^4^.

**Table 5:** Parameters used for simulation.

Simulation Parameter	Value
Sensor nodes	100–500 nodes
Sensing range of the nodes	100 –200 m
Sensor node’s initial energy	0.5 J
Sensing range	30 m
Data rate	250 kpbs
Size of packets	1024 bytes
Individual node’s sensing range	36 m
Maximum iterations	100

The effectiveness evaluation of the PHMM-FCSO is compared with existing models by using three important metrics namely.

- Fault node detection evaluation.
- QoS parameters of the network.

### Fault node detection evaluation

In this section the performance evaluation of fault node detection in the network using the throughput, fault detection accuracy, false positive and false alarm rates.

#### Fault Detection Accuracy

**Fig. 3:**
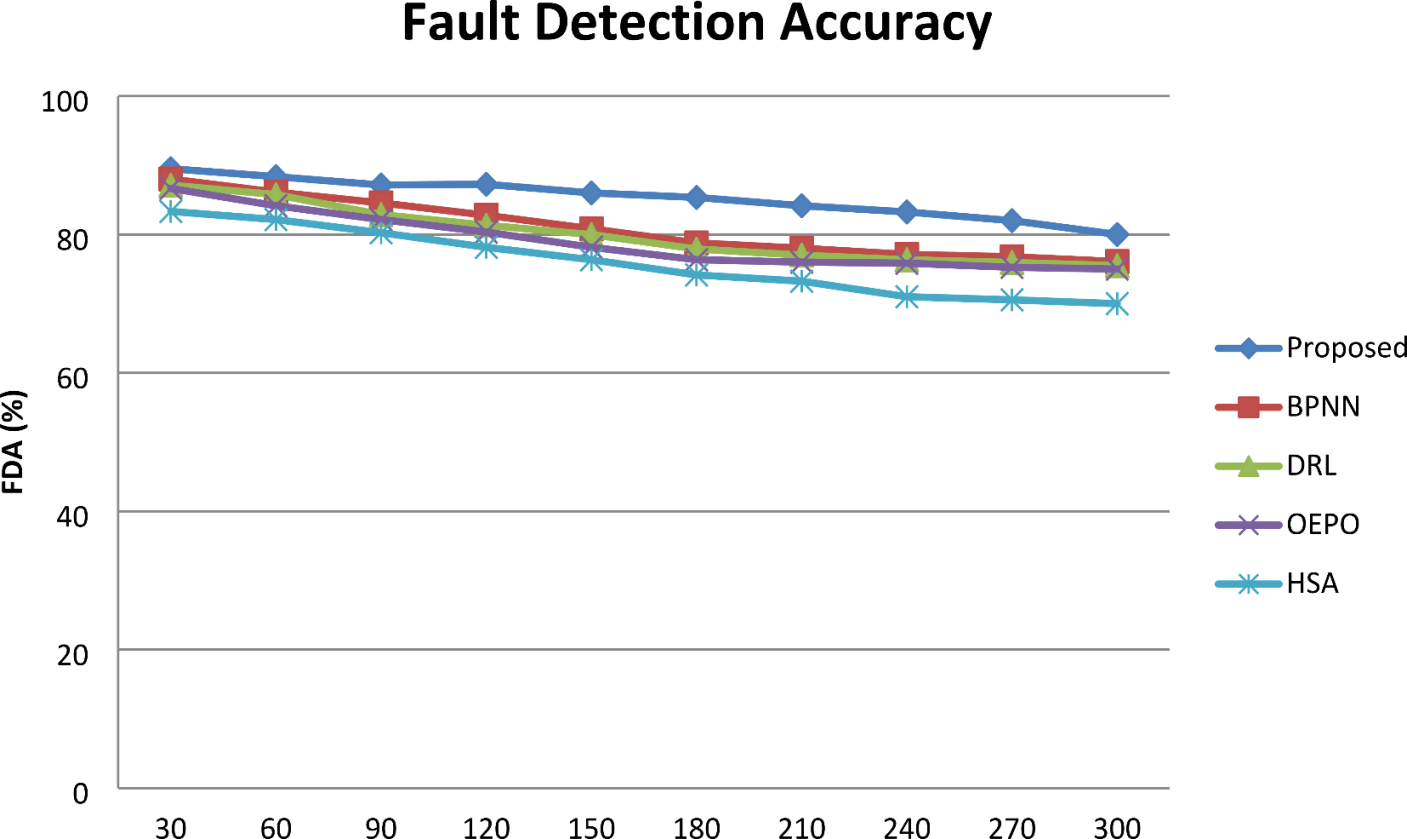
Comparative analysis of FDA.

Figure 3 shows the graphical representation of the fault detection accuracy. From the comparative analysis graph, increasing number of the sensor nodes involved in simulation results in a minimization of detection accuracy. However, for nodes 300, comparative analysis shows the resulting accuracy of 89.5% for the proposed system and 88.01% (BPNN), 87.12% (DRL) 86.46% (OEPO), 83.03% (HSA) for the existing systems, respectively. From this result, the accuracy of fault detection of PHMM-FCSO was observed to be comparatively better than the state-of-the art methods. The proposed work significantly detects faulty SNs in the network. The use of a suitable fault detection method, like PHMM in fault detection, is the primary factor in achieving this improved accuracy. The reason for enhanced accuracy is that the proposed model could identify both the hardware and software sensor faults; hence, the performance of detection accuracy is greater than the existing works. This in turn results in the improvement of fault detection accuracy using the proposed system by 1.5% compared to BPNN, 2.7% compared to DRL, 4.5% compared to OEPO and 7.3% compared to HSA.

#### False alarm rate (FAR)

**Fig. 4:**
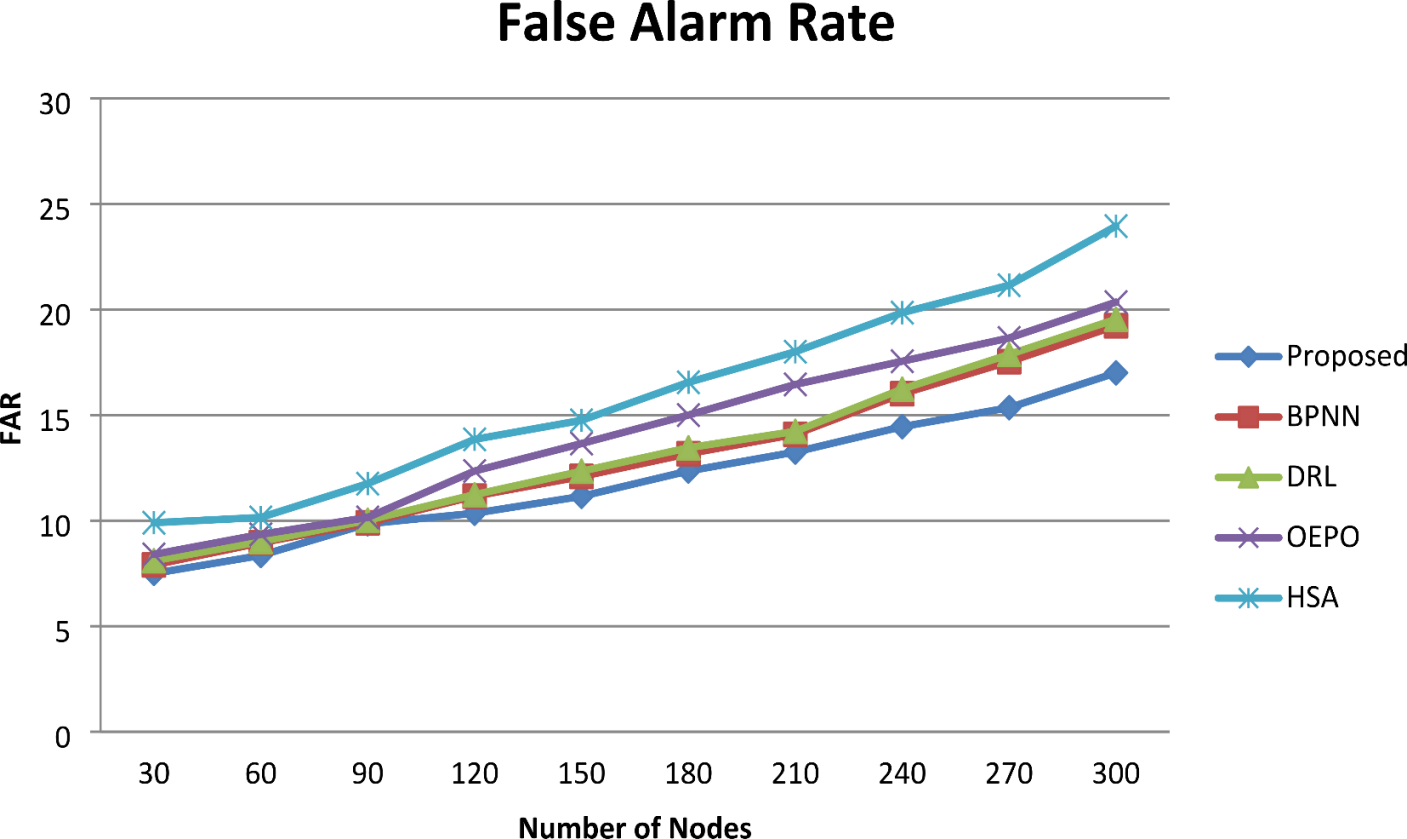
Comparative analysis of FAR.

Figure 4 shows the graphical representation of the fault alarm rate. From the comparative analysis graph, increasing the number of sensor nodes involved in simulation results in maximization of false alarm rate. However, for nodes 300, comparative analysis presents the resulting false alarm rate of 17% for the PHMM-FCSO and 18.23% BPNN, 19.54% (DRL), 20.35 (OPEO) and 23.95% (HSA) for the existing systems, respectively. From this result, the false alarm rate of the proposed system was observed to be comparatively better than the existing methods. The key reason for attaining this minimum False Alarm Rate is the proper implementation of an optimization algorithm like F-CSO. The Fuzzy based Chicken Swarm Optimization algorithm yields the minimum false alarm rate and it predicts the faulty sensor nodes very accurately. Moreover, the reason behind this greater performance is that F-CSO balances the exploration and exploitation in creating a new hierarchy and it enables the searching of the entire region to attain the optimal solutions.

#### False positive rate

**Fig. 5:**
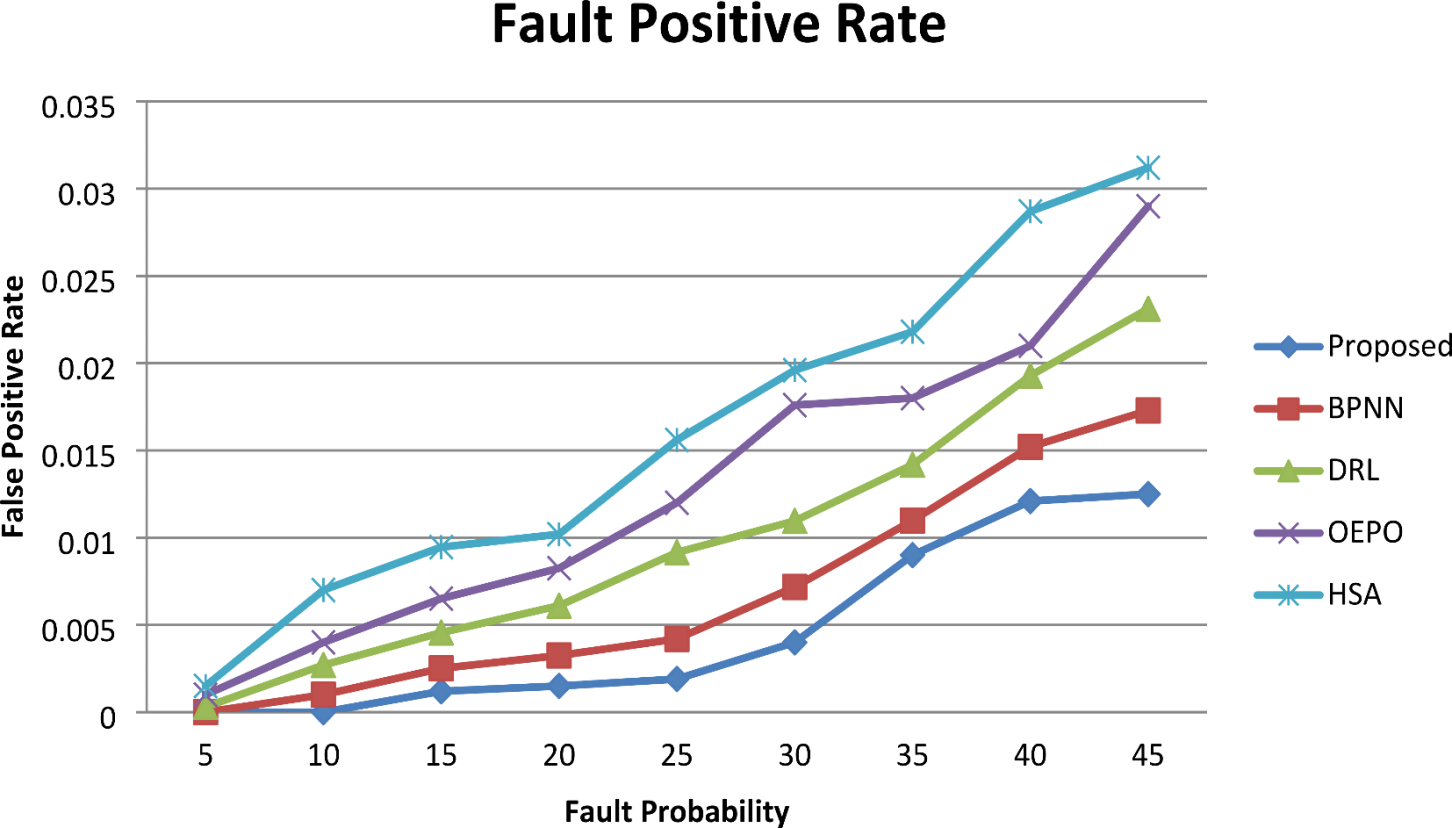
Comparative analysis of False Positive Rate.

Figure 5 shows the graphical representation of the fault positive rate. From the comparative analysis graph, increasing the number of faults percentage in sensor nodes involved in simulation results in a better false positive rate. However, the comparative analysis presents the resulting false positive rate, the fault probability for the PHMM-FCSO system is very low by comparing with all other existing works BPNN, DRL, OEPO, and HSA, respectively. From this result, the false positive rate of the proposed system was observed to be comparatively optimum than the existing methods. The proposed work achieves a very low rate of false positives than the other four existing works. The key reason behind attaining this minimum false positive rate is that the proposed work identifies both the hard and soft sensor faults.

#### Throughput

**Fig. 6:**
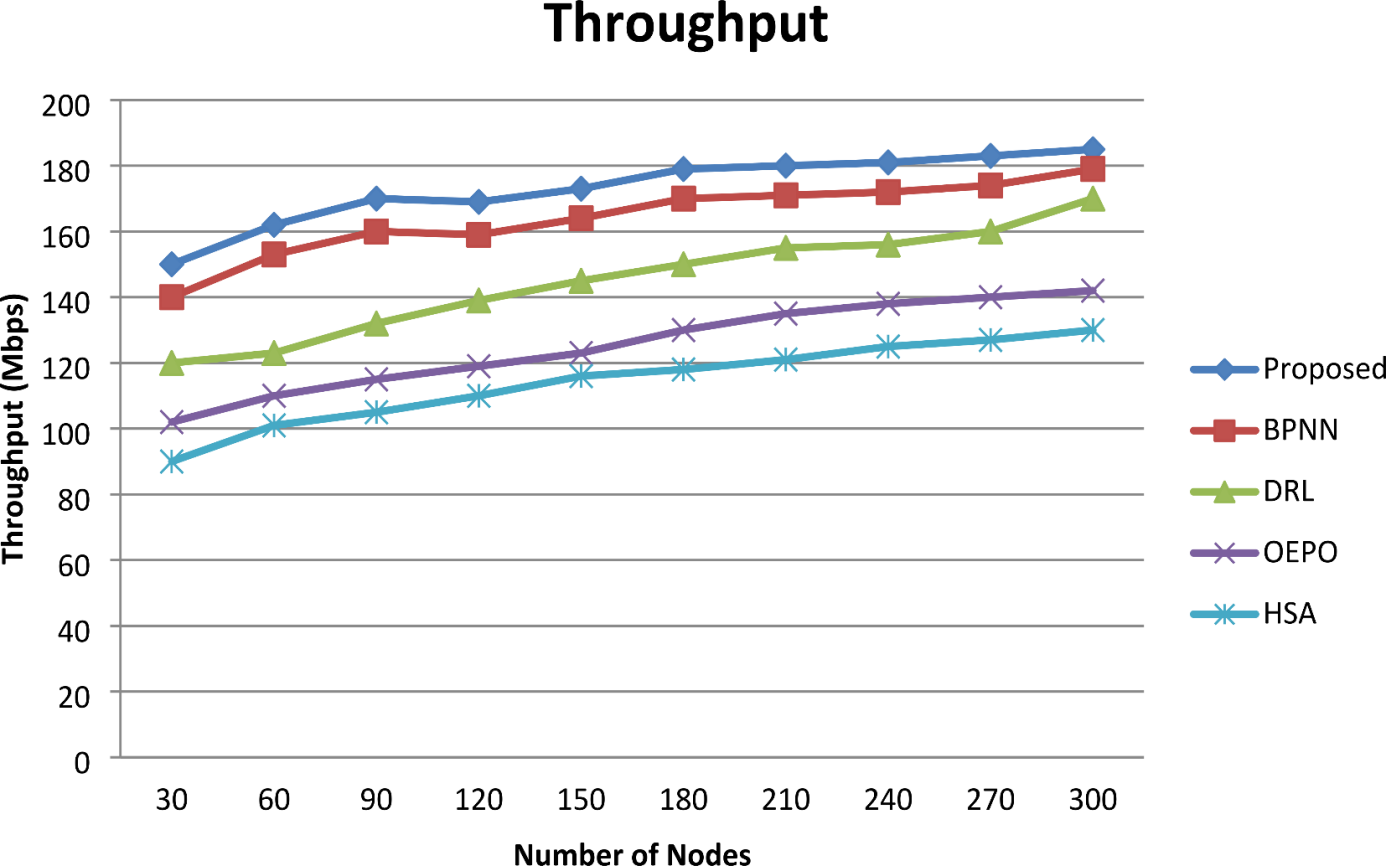
Comparative analysis of Throughput.

Figure 6 shows the graphical representation of the through. From the comparative analysis graph, increasing the number of sensor nodes involved in simulation results in maximization throughput. However, for nodes 300, comparative analysis shows the resulting better throughput for the proposed system compared with existing works OEPO, HSA, BPNN and DRL, respectively. As a result, the proposed system’s throughput was shown to be significantly superior to the existing approaches. The reason for achieving superior throughput is the implementation of the F-CSO model, which maintains the fault-free region of nodes during the data transmission.

### QoS parameters of the network

In this section the evaluation of fault node detection in the network using the Quality of Service parameters such as energy consumption, network lifetime and delay.

#### Average residual energy

**Fig. 7:**
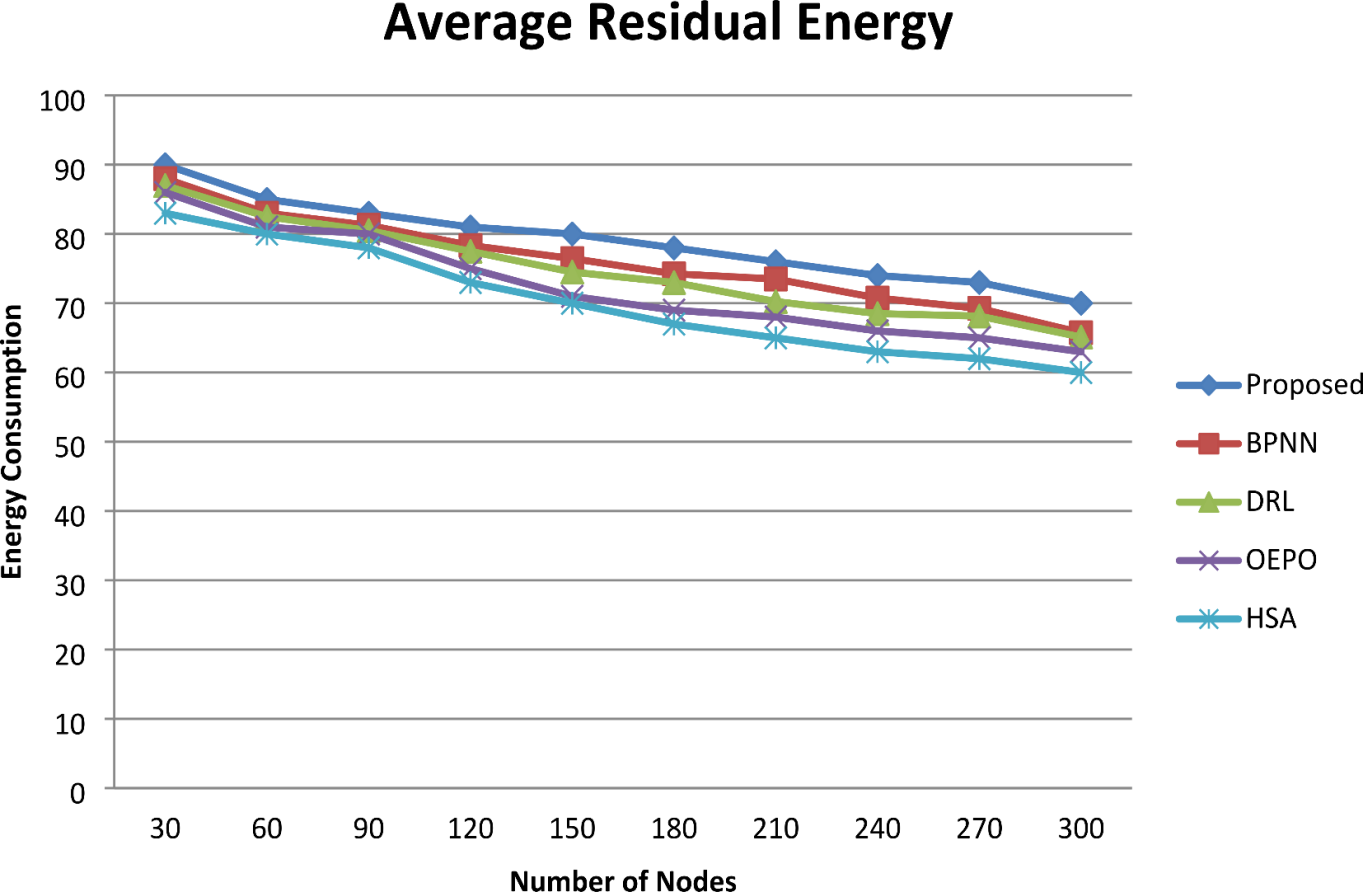
Comparative analysis of Energy.

Figure 7 shows the graphical representation of the average residual energy. From the comparative analysis graph, increasing the number of sensor nodes involved in simulation results in minimization of residual energy. However, for nodes 300, comparative analysis shows the resulting accuracy of 70.05% for the proposed system and 65.75% (BPNN), 65.03% (DRL), 62.96% (OEPO), 60.03% (HSA) for the existing systems, respectively. From this result, the remaining residual energy of the proposed system was observed to be comparatively optimum than the existing methods. Because the proposed work employs a robust fault management strategy and can detect the premature death of a sensor node. The proposed work avoids such premature death of sensor nodes at the earliest and battery unit failure. Moreover, the proposed work prolongs the lifetime of the network.

In the proposed system, the reason for the improvement in energy conservation is that the BS handles the fault diagnosis and localization while every sensor node presents its appropriate data to the BS for procession. The proposed fault detection and localization method progressively enhances the energy conservation of SNs, despite an initial processing overhead. This enhancement is achieved through the protocol’s precise detection of faulty nodes and optimization of network paths, which minimize energy waste from unsuccessful communication attempts and reduce unnecessary data retransmissions. The proposed protocol reduces energy-intensive procedures like retransmissions and dependence on faulty nodes by identifying faults early and making sure that traffic is routed through healthy nodes. This lowers the network’s overall energy consumption, increasing the SNs’ lifespan and making the initial computational load a fair trade-off for long-term energy efficiency.

#### Delay

**Fig. 8:**
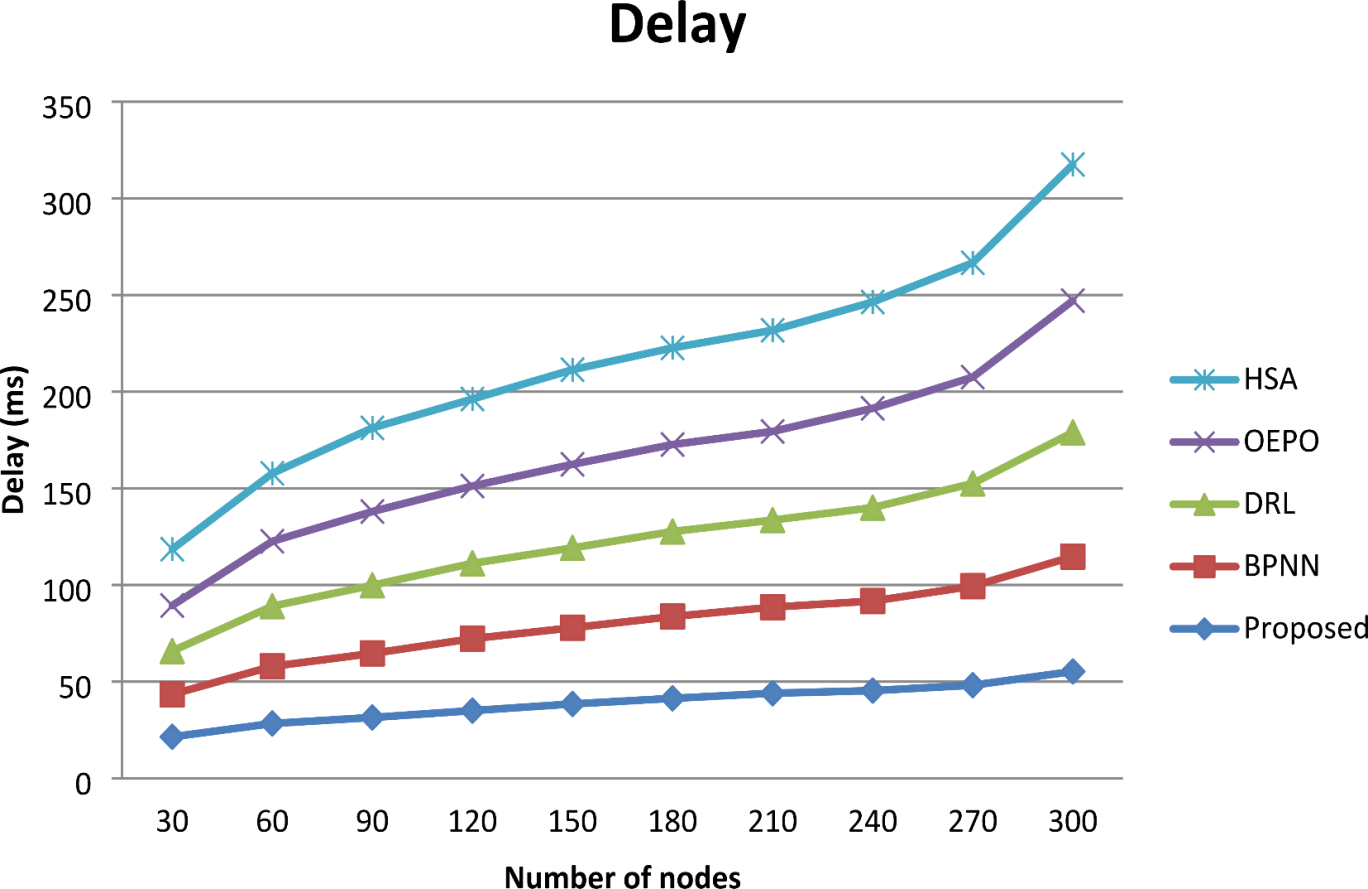
Comparative analysis of delay.

Figure 8 shows the graphical representation of comparative analysis of delay. From the comparative analysis graph, increasing the number of sensor nodes involved in simulation results in maximization of delay. However, for nodes 300, comparative analysis shows the resulting minimum delay is observed comparing the existing system. The reason for achieving optimum delay, in the proposed work is that the base station handles the fault detection phase while each sensor node provides its corresponding data to the region head for procession. In comparison to the other four existing works, this proposed work uses less energy and has a lower delay for fault detection. As a result, as nodes increased along with the probability of a fault, the performance of the proposed work was better than the existing works.

#### Network Lifetime

**Fig. 9:**
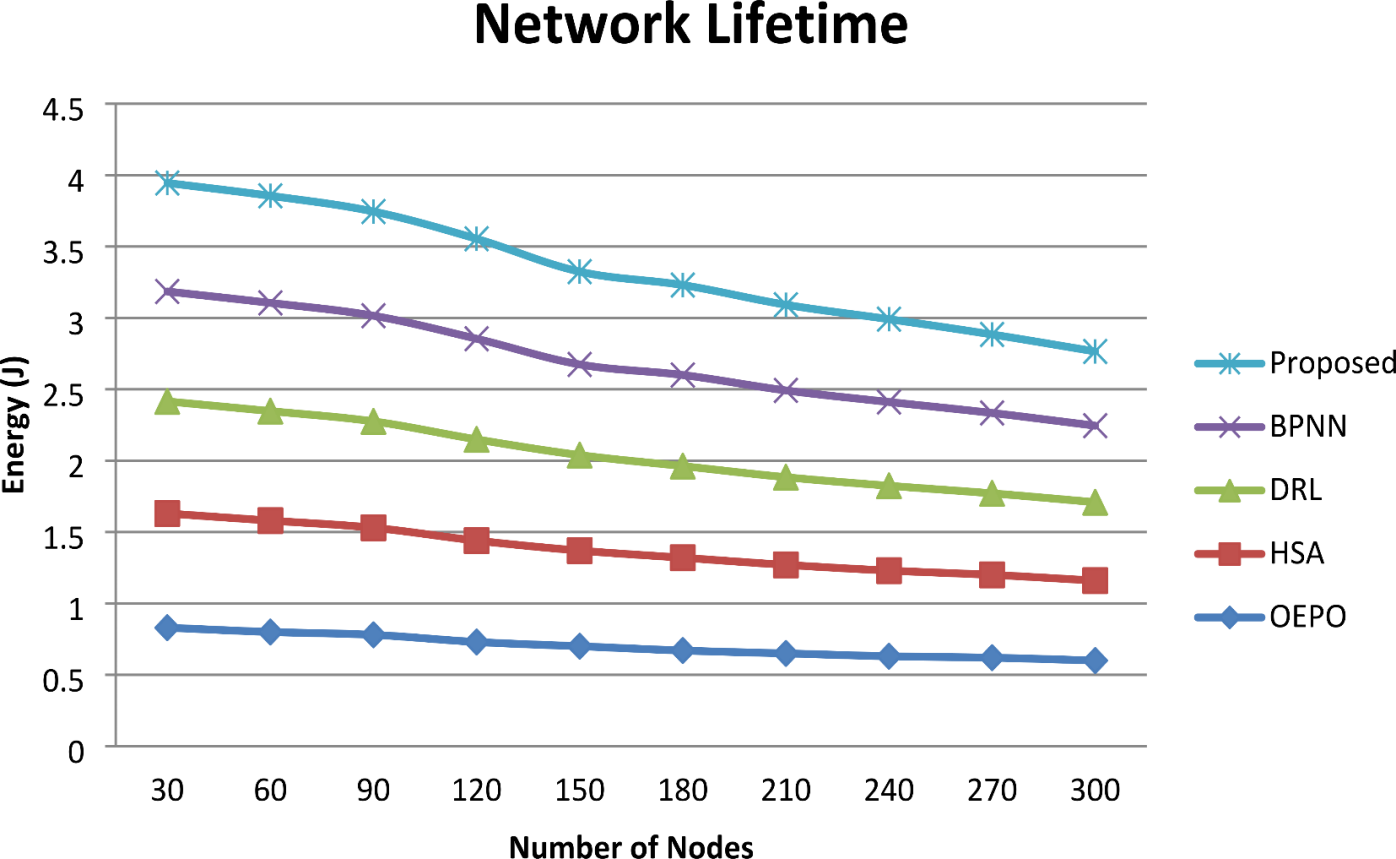
Comparative analysis of Network Lifetime.

Figure 9 shows the graphical representation of comparative analysis of network lifespan. From the comparative analysis graph, increasing the number of sensor nodes involved in simulation results in minimization of network lifetime. However, for nodes 300, comparative analysis shows the results of the proposed system was observed to be comparatively optimum than the existing methods. The proposed work considerably increases the network lifetime by maintaining neighbour node information to identify the faulty sensor nodes.

### Complexity analysis

To understand the effectiveness and performance of the proposed protocol, it is essential to analyze its theoretical complexity, especially regarding the fault node detection and localization in WSNs that utilize F-CSO with PHMM.

Computational Complexity of the PHMM: The complexity of the PHMM can be estimated by utilizing the number states k and number of steps T and complexity can be written as $O\left( {k^{2} \cdot \:T} \right)$.

Computational Complexity of the F-CSO: The time complexity of the F-CSO can estimated by utilizing the number of chickens CN, number of iterations IN, and dimension DN and the time complexity can be written as $O\,\left( {CN \cdot IN \cdot DN} \right)$.

## Conclusions and future work

An effective sensor node failure detection technique using the Poisson Hidden Markov Model (PHMM) and the Fuzzy based Chicken Swarm Optimization (F-CSO) is proposed in this work to handle these issues and achieve the best optimization in terms of QoS. Improved sensor node failure detection is provided by the suggested system, and as a result, better quality of service is achieved in terms of better fault detection accuracy, false positive rate, throughput, false alarm rate, energy consumption, network lifetime, and least latency. Additionally, the F-CSO offers improved localization following a network fault brought on by a sensor node. The NS2 simulator is used to implement the suggested task while using a variety of simulation parameters. According to the simulation results, the suggested work is more effective than the current state-of-the-art systems in terms of fault detection accuracy, falut positive rate, throughput, false alarm rate, delay, network lifetime, and energy consumption. In future development of the proposed protocol can be enhanced by advanced optimization approaches and other machine learning approaches to improve performance and longevity of the sensor networks.

## Data Availability

All data generated or analyzed during this study are included in this published article.
